# Trend of Smuggled Cigarette Consumption in Tehran in the Last Two Decades

**DOI:** 10.34172/aim.2022.71

**Published:** 2022-07-01

**Authors:** Gholamreza Heydari, Luk Joossens

**Affiliations:** ^1^Tobacco Prevention and Control Research Center, National Research Institute of Tuberculosis and Lung Diseases, Shahid Beheshti University of Medical Sciences, Tehran, Iran; ^2^Foundation against Cancer, Brussels, Belgium

**Keywords:** Illegal brands, Illicit cigarettes, Trend

## Abstract

**Background:**

Illicit tobacco trade is focused on Iran as a main target in the WHO’s eastern Mediterranean region. Serial studies of Cigarette Packs Survey with same method were conducted in Tehran between 2003 and 2015 to evaluate consumed smuggled cigarettes. This study as the fourth Cigarette Packs Survey is designed to indicate the trends of illicit cigarette trade in Tehran in the last two decades.

**Methods:**

A cross-sectional household study was carried out in early 2021 in Tehran on 3042 persons who smoked at least one daily cigarette for a year. The sampling method was like the sampling method used in three previous studies. Participants aged≥15 years were asked to reveal their current cigarette pack, which was either legal cigarettes (having governmental label); or illegal cigarettes (without governmental label).

**Results:**

The subjects included 2536 males (83.4%) and the mean age was 39.9±12.1 years; 1854 subjects (60.9%) showed foreign cigarettes and 1188 (39.1%) showed domestic cigarettes; 2705 (88.9%) consumed legal cigarettes and 337 (11.1%) consumed illegal cigarettes. Consumption of illegal cigarettes by gender showed greater use of smuggled cigarettes in males (11.7%vs 8.1%). No significant differences were seen based on the marital and educational status in terms of illegal cigarettes prevalence.

**Conclusion:**

Compare with previous studies, the trend of consumption of illicit cigarettes was decreasing in past two decades. This could be due to new regulation on monitoring cigarette distribution and changing illegal brands to legal as joint production.

## Introduction

 According to the literature on global illicit trade, illicit cigarettes were the difference between legal imports and legal exports.^1,2^ The majority of this difference was channeled into the illegal tobacco business, mostly provided from large-scale illicit. About 8% of global cigarette consumption was illegal in 1995.^3^

 Based on a report on cigarette illicit trade, about 11% of cigarette consumption was illicit in 2009: which was 16.8% in low-income countries, 11.8% in middle-income countries and 9.8% in high-income countries. About 657 billion cigarettes a year were consumed in these countries as total annual smuggled, which consisted of 533 billion in low- and middle-income countries and 124 billion in high-income countries.^4^

 Illicit tobacco trade targeted Iraq and Iran as two main states for marketing in the eastern Mediterranean region. Iran has a state-owned monopoly for tobacco, but foreign brands were highly demanded.^5^ According to unofficial reports from the government and the Iranian Tobacco Company, the prevalence of illicit tobacco products was estimated 20% and 8–10%, respectively. In 1998, a foreign tobacco company declared that 60% of the total market in Iran was illegal, while its market share (mainly Winston and Magna) was approximately 40% of total consumption. Serial studies of Cigarette Packs Survey with the same method were conducted in Tehran between 2003 and 2015 which indicated that 40%, 20.9% and 15.4% of smokers consumed smuggled cigarettes respectively.^6-8^ Those studies as household surveys were carried out through a questionnaire filled out by smokers self-reporting their cigarette packs to evaluate being legal or illicit. This present study was the fourth one done through the observation of smokers’ cigarette packs to find the trend of illicit cigarette consumption in Tehran for the past two decades.

## Materials and Methods

 Data were collected from the fourth cigarette pack survey to evaluate the illegal cigarette consumption in Tehran with the same method.^6-8^ This study was a cross-sectional household survey of smokers aged 15 and over in Tehran, conducted from September 2020 to February 2021. Smokers included who reported having smoked one cigarette a day for at least a year. The sample size formula was n = Z^2^ (p) (1-P)/d^2^ (with α = 0.05, d = 0.02, Z = 1.96 and *P* = 0.25) (with *n* = sample size, d2 half-length of confidence interval 0.02, α = type 1 error 0.05, Z 1-α/2 = 100 (1-α/2) percentile of normal distribution 1.96 and *P* = prevalence of illegal cigarette in the last study^8^ (0.25) was applied to all district populations of Tehran (Supplementary file 1, Table S1) to obtain a sample size of 1801. Considering the increasing population and to achieve more power, the design effect of 0.25 was added to the size that had 2294 with probability proportional to size. A random point in each of the 22 Tehran districts was selected and then proceeding left until the sampling quota of smokers had been interviewed.

 Persons aged 15 years and older were interviewed and their tobacco consumption, tobacco purchase information, as well as socioeconomic and demographic characteristics were collected. In particular, the price per cigarette or per pack was asked. Detailed brand information was used to identify the purchase/use of manufactured legal and illegal cigarettes.

 Table S1 shows proportional sampling in the populations of Tehran’s 22 districts. Data collection continued to the left or until the sampling quota of subjects had been interviewed. Interviewers asked the participants to show their current cigarette packs, which were categorized as follows: legal cigarettes which displayed special governmental labeling and illegal cigarettes which had no special governmental labeling; national cigarettes which had a Persian name; foreign cigarettes which had a foreign name.

 Frequencies of legal and illegal cigarette packs were reported. The chi square test was performed on the consumption of illegal cigarettes by sex and *t* test was performed no the consumption of illegal cigarettes based on age with the significance level < 0.05.

 Trend analysis as underlying pattern of behavior in a time series was undertaken within a formal regression analysis, as described in trend estimation.

## Results

 The subjects included 2536 males (83.4%) and the mean age was 39.9 ± 12.1 years; 56.6% of participants had a high school diploma, 74.5% were married and 40.8% were self-employed. The subjects daily paid US$ 0.2–3.2 (mean 0.8 ± 0.3) for their cigarettes. The cost of smuggled cigarettes was 3 times more than the legal products.

 Overall, 1854 subjects (60.9%) showed foreign cigarettes and 1188 (39.1%) showed domestic products (Table 1); 2705 (88.9%) consumed legal cigarettes and 337 (11.1%) consumed illegal cigarettes (Table 2). No significant differences were seen based on the marital and educational status in terms of illegal cigarettes prevalence. Generally, female smokers consumed foreign cigarettes more than male smokers (71.9% vs. 58.8%, *P* = 0.001) (Table 1).

**Table 1:** Frequency of Foreign Cigarette Consumption by Gender in Tehran in 2021

		**Brand**		**Total**
		**Domestic**	**Foreign**	
Gender	Male	1046 (41.2%)	1490 (58.8%)	2536 (100%)
	Female	142 (28.1%)	364 (71.9%)	506 (100%)
	Total	1188 (39.1%)	1854 (60.9%)	3042 (100%)

*P* = 0.001, Odds ratio for gender (male/female) = 0.667; 95% CI = 0.474, 0.939

**Table 2:** Frequency of Illegal Cigarette Consumption by Gender in Tehran in 2021

		**Brand**		**Total**
		**Legal **	**Smuggle **	
Gender	Male	2240 (88.3%)	296 (11.7%)	2536 (100%)
	Female	465 (91.9%)	41 (8.1%)	506 (100%)
	Total	2705 (88.9%)	337 (11.1%)	3042 (100%)

*P* = 0.02, Odds ratio for gender (male/female) = 1.800; 95% CI = 1.460, 2.219

 A statistically significant difference was found in consumption of illegal cigarettes by gender which showed more smuggled in males (11.7% vs 8.1%; *P* = 0.02) (Table 2).

 A statistically significant difference was found in consumption of illegal cigarettes by younger smokers (37.8 ± 11.4 vs. 40.1 ± 12.2 years) (*P* = 0.001) (Table 3) and foreign cigarettes by younger smokers (37.9 ± 11.6 vs. 43 ± 12.2 years) (*P* = 0.001) (Table 4). In the regression analysis, a downward trend was seen (Table 5).

**Table 3:** Comparison of Illegal Cigarette Consumption by Age in Tehran in 2021

	**Brand**	**N**	**Mean**	**Standard Deviation**
Age	Legal	2676	40.1	12.2
	Smuggle	330	37.9	11.4

*P* = 0.0001, Mean difference = 2.2, 95% CI: 0.88, 3.65.

**Table 4:** Comparison of Cigarette Brand Consumption by Age in Tehran in 2021

	**Brand**	**N**	**Mean**	**Standard Deviation**
Age	Domestic	1170	43.06	12.2
	Foreign	1836	37.93	11.6

*P* = 0.01, Mean difference = 5.1, 95% CI: 4.25, 5.99.

**Table 5:** Generalized Linear Models for Binary Data

**Years**	**Illicit Cigarettes**		**Odds Ratio [Exp (beta)]**	**95% CI for Odds Ratio**		***P*** ** Value**
	**Yes**	**No**		**Lower**	**Upper**	
2005	1210	1816	—	—	—	< 0.001
2009	322	1218	2.520	2.185	2.907	< 0.001
2015	328	1802	3.661	3.188	4.204	< 0.001
2021	337	2705	5.348	4.675	6.119	< 0.001

 Marlboro was the only smuggled cigarette brand (337 packs; 100%). No significant differences were seen among illegal cigarettes bought from the newsstands and supermarkets (*P* = 0.59).

## Discussion

**Figure 1:**
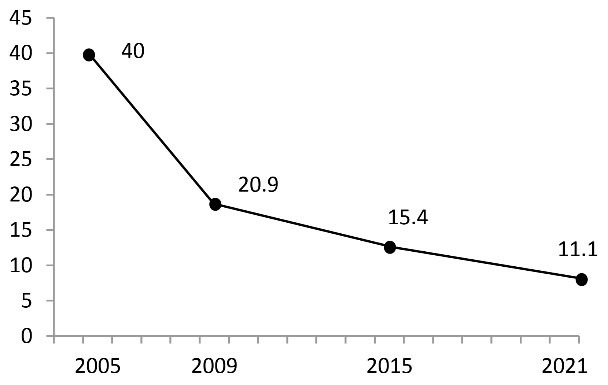
Trend of Consumption of Illicit Cigarettes in the Past Two Decades

 Smuggled cigarette consumption in Tehran was about 11% and less than the three previous pack surveys (Figure 1). It is notable that just one brand that had no legal contract with the government was the only smuggled cigarette in Tehran. Controlling and monitoring the legal distribution of cigarettes in retailing and banning street vending according to the regulations and legal joint production with some international brands may have caused a decrease in the trend of illicit cigarettes in Tehran during the last 20 years. Controlling borders and reducing transportations due to COVID-19 pandemic could be other reasons.

 High trend of smuggled cigarette consumption in the young adults and high prevalence of foreign cigarettes among women were also notable during the past two decades in Tehran ^9-11^ as the smoking trend was about 20% in males and 2% in females with no change in that time.^12,13^

 Based on the experiences from the literature,^14^ despite the reduction in consumption of smuggled cigarettes in Iran, more supervision is needed on controlling the sale of illicit cigarettes in order to achieve an extra reduction in the smoking trend and increase the government revenue.^15^

 Foreign brand cigarettes are in high demand in Tehran, particularly by youngsters. Various illicit versions of these brands are found in the city. In Iran, unlike other countries, illicit brands are legally sold beside the legal brands (national and foreign) in supermarkets and newspaper kiosks. Free sale of illicit cigarettes, for which no tax has been paid, brings about large taxation losses for the State. The Iranian Comprehensive National Tobacco Control Law (ICNTCL) has been approved by the Parliament. All vendors are required to have a tobacco sale license since June 2009 but some delay has occurred in the implementation of this law due to administrative turmoil. As a result, most vendors continue to sell the illicit alongside legal tobacco products. At first, it was supposed to provide guidelines of the article 7 (related to retail licensing of tobacco sale) of ICNTCL by August 2019 for methods of tobacco supply and sale. By beginning of 2020, a better situation will be achieved by approval of the guidelines and licensing of sale (see http://tpcrc.sbmu.ac.ir/index.jsp?fkeyid=&siteid=188&pageid=6701).

 In conclusion, the trend of consumption of illicit cigarettes has been decreasing in the past two decades. This could be due to new regulations on monitoring cigarette distribution, which included banning street trade, and tracking and tracing smuggled cigarettes. These might have controlled the illicit tobacco trade.

## Supplementary files

Supplementary file 1 contains Table S1.
