# Supplementary material for: Trend of Smuggled Cigarette Consumption in Tehran in the Last Two Decades
Source: Arch Iran Med. 2022 Jul 1;25(7):428–31. doi: 10.34172/aim.2022.71 (PMC11904283; doi:10.34172/aim.2022.71)
Supplement: Supplementary file 1 — contains Table S1. [file aim-25-428-s001.pdf]

Supplementary file 1

Table S1. Dispersion of Tehran Population and Proportional Sampling based on Tehran's 22 Districts Population Survey on 1396 (http://amarista.ir/content/statistics/385/).

District	Family	Male	Female	Total	Proportion (%)	N
1	141426	216011	223456	439467	5.3	123
2	205883	312830	320087	632917	7.7	179
3	106046	148621	165491	314112	3.8	88
4	270948	429903	431377	861280	10.6	243
5	255333	391297	402453	793750	9.7	223
6	73212	110751	119229	229980	2.8	65
7	108728	150025	159720	309745	3.8	88
8	125757	185515	192603	378118	4.6	106
9	50591	80744	77772	158516	1.9	45
10	104720	150050	152802	302852	3.7	86
11	98556	142877	146007	288884	3.5	81
12	76628	122141	118579	240720	2.9	68
13	89582	136706	139321	276027	3.4	79
14	153649	244122	240211	484333	5.9	138
15	192610	325313	313427	638740	7.8	180
16	89928	144578	143225	287803	3.5	81
17	75872	125364	123225	248589	3	69
18	115151	199623	191745	391368	4.7	108
19	70349	124481	119869	244350	3	69
20	104275	170859	170002	340861	4.1	93
21	50381	82014	80667	162681	2	46
22	38106	65476	63482	128958	1.5	36
Total	2597731	4059301	4094750	8154051	100	2294
